# A web-based information system for a regional public mental healthcare service network in Brazil

**DOI:** 10.1186/s13033-016-0117-z

**Published:** 2017-01-03

**Authors:** Vinicius Tohoru Yoshiura, João Mazzoncini de Azevedo-Marques, Magdalena Rzewuska, André Luiz Teixeira Vinci, Ariane Morassi Sasso, Newton Shydeo Brandão Miyoshi, Antonia Regina Ferreira Furegato, Rui Pedro Charters Lopes Rijo, Cristina Marta Del-Ben, Domingos Alves

**Affiliations:** 1 Interunit Bioengineering Postgraduate Program, University of São Paulo, São Paulo, Brazil; 2 Center of Information and Informatics in Health, Ribeirao Preto Medical School, University of São Paulo, São Paulo, Brazil; 3 Department of Social Medicine, Ribeirao Preto Medical School, University of São Paulo, São Paulo, Brazil; 4 Community Health Postgraduate Program, Ribeirao Preto Medical School, University of São Paulo, São Paulo, Brazil; 5 Department of Psychiatry and Human Sciences, Ribeirao Preto College of Nursing, University of São Paulo, São Paulo, Brazil; 6 School of Technology and Management, Polytechnic Institute of Leiria, Leiria, Portugal; 7 Department of Neuroscience and Behaviour Science, Ribeirao Preto Medical School, University of São Paulo, São Paulo, Brazil

**Keywords:** Health information systems, Mental health, Public health informatics, Health management, Health networks

## Abstract

**Background:**

Regional networking between services that provide mental health care in Brazil’s decentralized public health system is challenging, partly due to the simultaneous existence of services managed by municipal and state authorities and a lack of efficient and transparent mechanisms for continuous and updated communication between them. Since 2011, the Ribeirao Preto Medical School and the XIII Regional Health Department of the Sao Paulo state, Brazil, have been developing and implementing a web-based information system to facilitate an integrated care throughout a public regional mental health care network.

**Case presentation:**

After a profound on-site analysis, the structure of the network was identified and a web-based information system for psychiatric admissions and discharges was developed and implemented using a socio-technical approach. An information technology team liaised with mental health professionals, health-service managers, municipal and state health secretariats and judicial authorities. Primary care, specialized community services, general emergency and psychiatric wards services, that comprise the regional mental healthcare network, were identified and the system flow was delineated. The web-based system overcame the fragmentation of the healthcare system and addressed service specific needs, enabling: detailed patient information sharing; active coordination of the processes of psychiatric admissions and discharges; real-time monitoring; the patients’ status reports; the evaluation of the performance of each service and the whole network. During a 2-year period of operation, it registered 137 services, 480 health care professionals and 4271 patients, with a mean number of 2835 accesses per month. To date the system is successfully operating and further expanding.

**Conclusion:**

We have successfully developed and implemented an acceptable, useful and transparent web-based information system for a regional mental healthcare service network in a medium-income country with a decentralized public health system. Systematic collaboration between an information technology team and a wide range of stakeholders is essential for the system development and implementation.

## Background

Brazil is a rapidly developing middle-income country with the world’s fifth largest population. To meet specific regional healthcare needs for comprehensive, universal preventive and curative care of this large and diverse society, a unified and decentralized public healthcare system was created in Brazil in 1988 [1]. Since then there has been rapid scaling up of integrated care approaches to public healthcare provision [1]. However, a regional integration between services, including the mental healthcare system, is still limited and extremely challenging; mostly due to decentralization of health service structures and a complex private–public health care mix [1]. The development of the mental health services network began in the 80s and accelerated in recent years, including the expansion of community services and the reduction of beds in psychiatric hospitals. This rapid scaling up of a community-based mental healthcare approach has led to an urgent need to develop new, feasible integration strategies for mental health services in Brazil [2, 3]. Low and middle-income countries have successfully implemented web-based systems to integrate health services information, but not for mental health service networks [4].

Since the mid-90s, a gradual expansion of the mental health services network has occurred in the XIII Regional Health Department (XIII RHD) of the Sao Paulo State, including 26 municipalities with an overall population of approximately 1,400,000 inhabitants. This expansion has involved local and regional community-based services, outpatient clinics, psychiatric hospital wards and psychiatric wards in the general hospital of the Ribeirao Preto Medical School of the University of Sao Paulo (RPMS-USP) [5, 6].

The regional health committee that comprises state and municipal health authorities, established a mental health advisory group [7], as permitted by the enactment of the Brazilian health law legislation on that matter. The committee is managed by the state government, municipalities and the RPMS-USP. Amongst its statuary activities are monthly meetings, with representatives of all public mental health services in the region during which participants propose standards for the operation of mental health services, including guidelines for the coordination between hospital and community-based care.

Since 2004, the demand for mental health care has progressively increased in the region of the XIII RHD, resulting in longer waiting time for treatment and increasing difficulties with coordination of care between the services [5, 6, 8]. A major underlying problem was a rapid updating of information about the exact structure of the system and its flow. Another fundamental problem was an absence of suitable health information system technology in healthcare services (e.g. computers with internet connectivity and updated web browsers), especially in small municipalities. Consequently, information sharing and access within the public mental health service network were inadequate, lacking information on psychiatric beds availability, situation of the request lists for hospitalization, referrals, appointments, follow-ups and management reports.

To facilitate integration of the information and processes of care, the regional state health authority, with the support from municipal health authorities and the mental health advisory group, proposed in 2011 the development of a computerized information system, tailored to the specific characteristic and needs of the regional mental health care services network [9, 10].

## Case presentation

Following the request of regional health authorities, the Center of Information and Informatics in Health (Portuguese: “Centro de Informação e Informática em Saúde”—“CIIS”) of RPMS-USP obtained funding from the Secretary of Health of Sao Paulo state to develop the computerized system for the regional public mental health care service network. A socio-technical approach was used, in which the potential users actively collaborated with technical developers during the development and implementation of the computerized information system, through strategies, such as [11, 12]: (a) frequent meetings with different groups of stakeholders, both separately and jointly, formally invited by the regional mental health advisory group; (b) detailed discussions of requirements to be addressed by the new system, according to the needs and values of each group of stakeholders; (c) a definition of an operation model of the computerized system that meets a set of requirements of each group of stakeholders and implies the minimum required change in the working process of each type of service; (d) a definition of the minimum human resources needed (including system user training) and the minimum equipment needed for the adequate functioning of the system, considering the reality of the different services (including the capacity of municipal and regional managers to make new investments) and (e) a definition of a feasible process of maintenance and improvement of the computerized system considering the needs, values and resources of each group of stakeholders and the different services.

The mental health advisory group and the CIIS team made the initial decision to involve a wide range of professionals from different services and not involve patients and/or their family members, given that only the former group will be the system users. The invited health service managers, health professionals, municipal and regional health secretariats and judicial authorities decided to initially implement a web-based system to improve the process of requests and authorizations for hospital admissions and discharges within the network, hosted in a server of the RPMS-USP and using only free, nonprofit software and web address’ service. To achieve this goal, the system should facilitate the matching of each patient with the most appropriate hospital or community-based service available by detailed sharing of patient information between services. It was decided by the committee that the system would aim to provide transparent and real-time information through performance indicators for each service and the whole network, such as number of requests for hospitalization, waiting time for hospitalization, types of hospitalization and discharges processed, length of stay, number of occupied beds and psychiatric diagnosis of admitted patients.

The technical developers and stakeholders established that there were computers with internet access and human resources available in each health care service that could be incorporated in the new system, if an initial training and ongoing technical support by the RPMS-USP were provided. The following software was adopted: MySQL Database Management System and NetBeans platform framework. Web technologies such as hypertext pre-processor (PHP), hypertext markup language (HTML), cascading style sheets (CSS) and javascript were used.

Following the end of the developmental phase, the involved stakeholders and administrative staff of the health care services completed training sessions. A system’s pilot test was carried out in August–October 2012 and subsequently it entered in the operational stage. Moreover, appointed members of the regional mental health advisory group and the information technology team formed a monitoring group to perform monthly evaluations and improvements of the system. They have decided that the system’s capacity to achieve the aims for which it had been created would be assessed by the total number of its users, the total number of system’s access and through feedback of the system users (including verbal descriptions and a system survey).

**Fig. 1:**
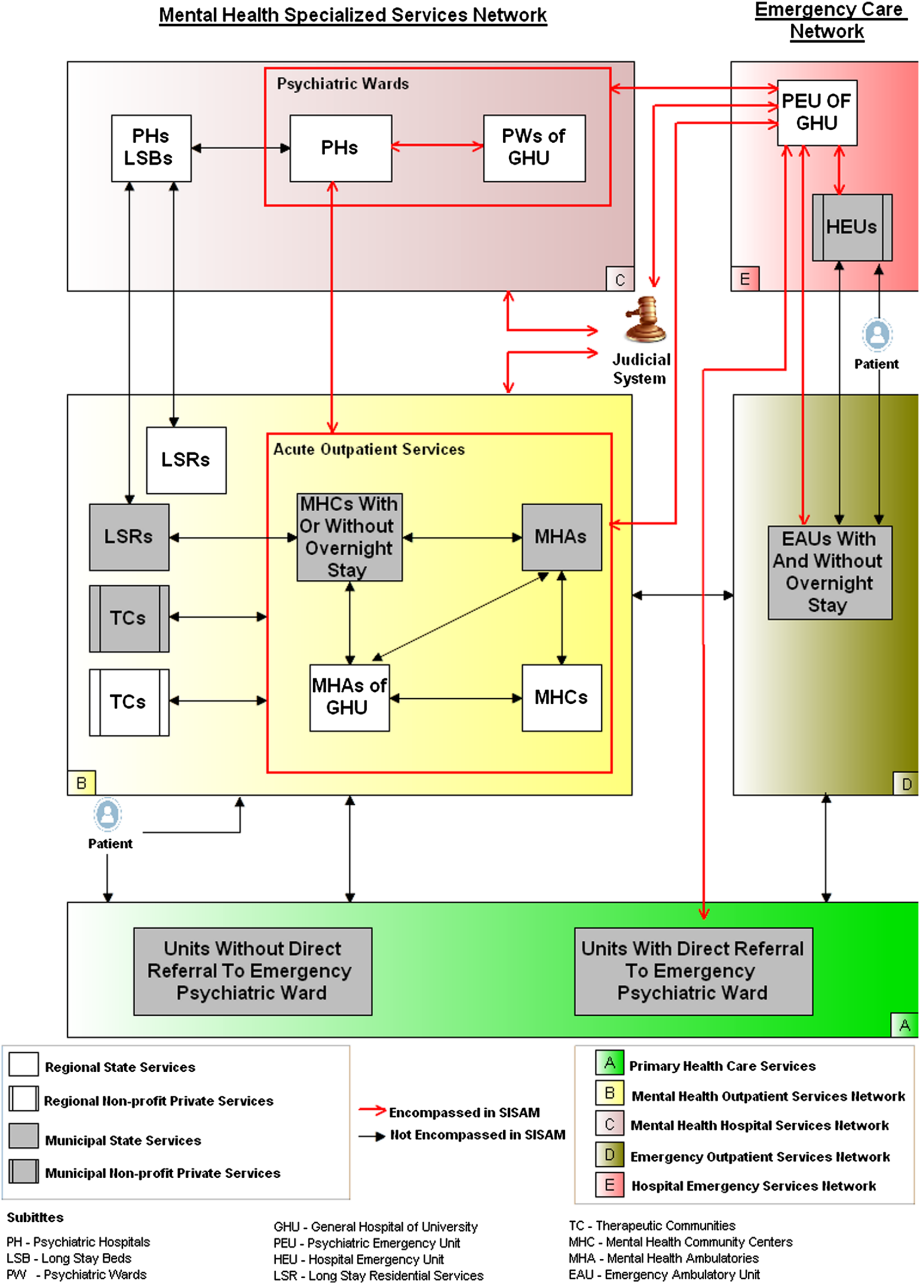
Structure and flow of the mental healthcare service network of the XIII Regional Health Department. *EAUs* emergency ambulatory units, *HEUs* general hospital emergency units, *LSRs* long stay residential services, *MHAs* mental health ambulatories, *MHAs of GHU* Mental Health Ambulatories of General Hospital of University of Sao Paulo, *MHCs* Mental Health Community Centers, *PEU of GHU* Psychiatric Emergency Unit of General Hospital of University of Sao Paulo, *PHs* Psychiatric Hospitals, *PHs LSBs* Psychiatric Hospitals Long Stay Beds, *PWs of GHU* Psychiatric Wards of General Hospital of University of Sao Paulo, *TCs* therapeutic communities

A series of discussions with the potential service users led to a consensus on the exact structure and flow of the public mental healthcare service network (MHSN) in the region of XIII RHD (Fig. 1). Whilst the stakeholders recognized serious difficulties in sharing information between services within the same municipality or services managed by the state, they concluded that each service follow the federal standards of functioning.

The red arrows represent possible ways of patient referrals encompassed in the developed system, including judicial requests, whereas the black arrows represent possible ways of patient referrals that have not yet been incorporated in the system. Additionally, while the orange boxes portray regional services, which attend patients from all municipalities of the XIII RHD, the blue boxes portray municipal services that only attend local population.

The MHSN consists of mental health specialized services network (MHSSN), an emergency care network (ECN) and primary health care services. The MHSSN is divided into: a hospital service network (acute and long-stay wards in psychiatric hospitals and acute psychiatric wards of the General Hospital of University of Sao Paulo—GHU) and an outpatient service network (community mental health centers with or without overnight stay; mental health ambulatories, including those in the GHU; therapeutic communities for alcohol and drug users and long-stay residential services). The ECN consists of: an emergency hospital service network (general hospital emergency units and a psychiatric emergency unit of the GHU) and an emergency outpatient service network (emergency ambulatories units with or without overnight stay).

The developed web-based system was entitled ‘Sistema de Informação em Saúde Mental do XIII Departamento Regional de Saúde do Estado de São Paulo’ (‘SISAM-13’; English: ‘Mental Health Information System of the XIII RHD’) [10]. Firstly, it allows: registering, searching and updating information for all patients (both personal and clinical data, including appointments, follow ups, requests for hospital admissions, hospital admissions and referrals); and management of reports for each service and the network as a whole. Secondly, it enables monitoring in real time with transparency of the waiting lists for hospital admissions and the availability of psychiatric beds. Finally, the system allows users to provide feedback, suggestions and request help directly from the information technology team. Table 1 shows the SISAM-13 information fields (patient socio-demographic information, service administrative information and patient medical history), completed using a list of possible and free response answers.

**Table 1:** The SISAM-13 information fields

Information type	Details
Patient sociodemographic information	
Patient information	
Patient’s name	
Mother’s name	
Date of birth	
National identification	Identity card number
Taxpayer registry identification	Individual taxpayer registry identification number
Public healthcare identification	National health card number
Gender	Male, female, transsexual
Address	Street, district, state, city, zip code
Telephone	Residential, mobile phone, work
Race/ethnicity/skin colour	Black, white, yellow, indigenous, brown
Marital status	Married, single, divorced, widow/widower
Living arrangements	Living with a partner, parents, children, other family members, acquaintances/friends, alone, other
Occupation	
Spouse, emergency contact, next of kin or carer’s information	
Name	
Relationship	Spouse, brother/sister, father/mother, other
E-mail	
Service administrative information	
User’s information	
Name	
Gender	Male, female, transsexual
Type of professional council	Regional Council of Medicine, nursing, psychology, social service, physiotherapy, nutritionist, lawyers, other
Professional registration number	
Issuing authority	
E-mail	
Telephone	Residential, mobile phone, work
System registration date	
Login	Username, password
Current place(s) of work	
Type of the system access permission	Psychiatrist, physician, resident physician, others health professional, shared use within the service, service manager, service secretary, system administrator, judge
Service’s information	
Service	Name, Address, State, Zip Code, City
Type of health service	Primary health care, mental health outpatient, mental health hospital, emergency outpatient, emergency hospital service
Regional Health Department	Name
Contact	Email, telephone
Number of beds	
Type of bed	Male, female, both
Hospital ward	
Patient’s medical history information	
Outpatient care	
Service	Name
Referral date	
Type of referral	Scheduled new case, scheduled return, counter-reference, emergency/clinical intercurrence, workshop
Professional(s) involved in the patient´s care	Physician, social worker, nurse, nursing technician, dentist, physiotherapist, psychologist, physical educator, speech therapist, pharmacist, occupational therapist, nutritionist, other
Type(s) of care	Individual, group, family care, home visit, workshops, psychosocial rehabilitation, other
Reference and counter-reference	
Type	Reference/counter-reference
Date	
Responsible healthcare professional	
Service/municipality of origin	
Destination service/municipality	
Motive	
Requests for hospitalization	
Requesting health professional	
Service/municipality of origin	
Hospital/municipality service	
Overnight stay at the service of origin	Yes/no
Message history between health professionals	
Request for hospitalization history	
Name of the judge	Judiciary request for information
District Attorney	Judiciary request for information
Applicant	Judiciary request for information
Judicial district	Judiciary request for information
Case number	Judiciary request for information
Number of the judicial process	Judiciary request for information
Judicial order number	Judiciary request for information
Compulsory indication	Treatment for chemical dependency, treatment for mental disorder, other
Judicial injunction	Judiciary request for information
Hospitalization	
Hospital/municipality	
Medical record number	
Bed number	
Responsible healthcare professional	
Hospitalization date	
Type of hospitalization	Determined by a judicial authority, determined by a psychiatrist without patient’s consent, determined by a psychiatrist with patient’s consent
Service of origin	Name, Municipality
Requesting health professional	
Date of discharge	
Type of discharge	By a psychiatrist, at the request of the patient or his/her family, administrative (ex. due to patient’s misbehaviour), escape, death, inter-hospital transfer in the network or transfer to other medical specialities
Name of the professional responsible for discharge	
Inter-hospital transfer destiny	
Motive for inter-hospital transfer	
Patient clinical information	
Outpatient care	
Primary diagnosis	According to the ICD-10
Other diagnosis	According to the ICD-10
Summary	Summary of the patient’s consultation/activity/workshop
Request for hospitalization	
Motive for request	Abstinence from use of psychoactive substances, psychomotor agitation, self-harm/hetero-aggressive behaviour, delirium tremens, suicidal ideation, psychoactive substance intoxication, judicial request, first psychiatric outbreak, severe depressive illness, maniac outbreak, other
Hypertension	Yes, no, no information
Diabetes	Yes, no, no information
Infectious disease	Yes, no, no information
Trauma	Yes, no, no information
Respiratory problems	Yes, no, no information
Sequelae of cerebrovascular accident	Yes, no, no information
Epilepsy	Yes, no, no information
Others comorbidities	Name
Medical exams	
Medication	
Initial diagnostic hypothesis	According to the ICD-10
Other diagnostic hypothesis	According to the ICD-10
Treatment modality	Intensive, non-intensive, semi-intensive
Hospitalization	
Motive for hospitalization	Abstinence from use of psychoactive substances, psychomotor agitation, self-harm/hetero-aggressive behaviour, delirium tremens, suicidal ideation, psychoactive substance intoxication, judicial request, first psychiatric outbreak, severe depressive illness, maniac outbreak, other
Primary diagnosis	According to the ICD-10
Other diagnosis	According to the ICD-10
Discharge primary diagnosis	According to the ICD-10
Discharge other diagnosis	According to the ICD-10
Comorbidities	
Initial medication	
Discharge medication	
Medical exam results	
Summary of clinical history	
Treatment	

*ICD-10* the International Statistical Classification of diseases and related health problems, Version 10

**Fig. 2:**
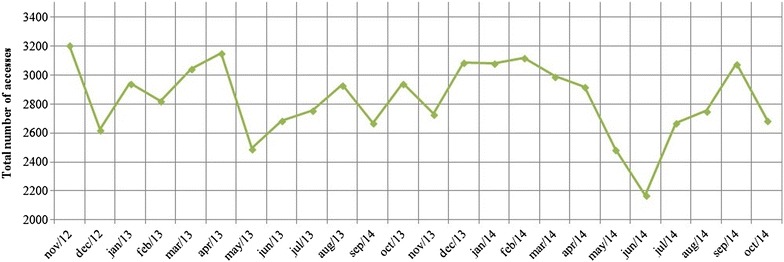
Total number of accesses per month to the ‘Mental Health Information System of the XIII RHD’

From November 2012 to October 2014, the system recorded 4271 patients, 480 professionals and 137 services. As shown in Fig. 2, a mean number of 2835 system accesses per month (range of 2172 to 3204) occurred and the smallest number was counted in June 2014, which is probably related to the modification of the initial address of the system (which became a paid service in June 2014). In addition, the system contains information on requests for hospitalizations and hospitalizations performed by each service. For example, during the study period, the SISAM-13 registered: 2391 requests and 1437 hospitalizations for the psychiatric hospital of Ribeirao Preto; 1732 requests and 732 hospitalizations for the psychiatric emergency service; and 541 requests and 419 hospitalizations for the psychiatric ward in the general hospital, data that can be divided in other types of information that can be provided.

During that period of 2 years, the monitoring group found through conversations with the stakeholders that the majority of users considered that the objectives of the web-based system had been met. Information sharing has become easier and faster, facilitating discussions and decisions about the type of care each patient needed. Municipalities that lacked any information system now use the SISAM-13 to assess information on referrals to hospitalization services [10]. The system generates hospital indicators that are important for decision making processes of the advisory group whose goal is to upgrade the existing services and create new ones. The main difficulties encountered during the system development and implementation included the conciliation of different interests from the stakeholders and the resistance of some health care professionals to use health information systems due to unfamiliarity and reservations about its security.

From September 2013 to April 2014, a survey was made available in the main menu of the system in order to verify the feasibility of the system. Twenty-six people participated in the survey: 80.76% were satisfied with the improvement of their working environment; 69.23% were satisfied with the navigability of the system; 84.61% were satisfied that the system conforms to their requirements; 80.76% were satisfied with the accuracy and consistency of the information; and 80.76% were satisfied with the improvement of patient care. The questionnaire included an open-ended question requesting that users add any comments they might have regarding the SISAM-13. The criticisms made to the system were related to the following topics: (a) difficulties in access or maintaining access to the system through the internet; (b) difficulties for the same professional to access the information about hospitalization requests from different patients within the SISAM-13 (the user had to return to the home page to restart the entire process for each patient); (c) a lack of an intelligent search engine by patient name, with the exact match option only being available; (d) a lack of “report” option with graphical displays; (e) a lack of possibility to make a new request for hospitalization without having to cancel the previous request and (f) a lack of pop-up notifications about received communication. The monitoring group used this feedback to continue improving the system.

Semi-structured interviews were conducted in 2016 as part of an ongoing qualitative study aiming to understand the daily practice of health community services in care of primary care patients with coexisting chronic physical health problems and depression or anxiety (including the use of computerized information systems) [13]. To date sixty primary care and mental health professionals were interviewed. Thematic analysis of the data suggests that the SISAM-13 facilitates psychiatric hospitalization by both general practitioners and community mental health specialists:

*“I can forward (for hospitalization) […] I put [a patient] in the system, which is the SISAM, a patient with psychotic outbreak, suicide attempt, self*-*harm. There’s a place I can do that and see where he has gone”.* (General Practitioner)

*“The SISAM is working well. We are using it more to see or to request hospitalizations and all patients who are now going through consultations I register with the SISAM”.* (Community mental health service manager 1)

The SISAM-13 also plays an important role in information sharing and subsequently enhancing continuity of care:

*“The SISAM that also makes it easier for us, we can see if there is a patient from the outpatient clinic that is waiting for a vacancy and talk about that patient or family member looked after by us, finally we can make this exchange [of information].* ”(Community mental health service manager 2)

*“A patient escaped from a [psychiatric] hospital and he came here […] I knew that I had requested his hospitalization by the SISAM in this psychiatric hospital, […] she should have come with a [discharge] conuter*-*reference. He came without it. This is how we found out [that he escaped]”* (Community mental health service manager 3)

A Ph.D. project will be conducted in 2017 [14] involving semi-structured interviews and focus groups, with various users of the SISAM-13 on their experiences with the system a during 5-year period of its implementation.

Hearing experiences and addressing needs of a wide range of stakeholders in the process of development and implementation is challenging [15], but it was necessary to achieve this first Brazilian web-based system that meets specific service needs, integrate public mental health care service information and is currently being used in daily clinical practice. Therefore, it is important to understand and match different drives and interests of the stakeholders [16]. While health care professionals are interested in the system’s capacity to ease the access to detailed patient information necessary for clinical decision making and the process of admission and discharge, managers and health authorities are driven by the system’s capacity to inform allocation of health care resources.

The large numbers of actions performed by the users of the SISAM-13 and the positive feedback received confirmed its acceptability, feasibility and utility [9–12]. This enhanced networking between services facilitates an integrated regional mental health care and can be expected to make an important contribution to the improvement of the quality of patient care.

## Conclusion

We have explored the local context, and specifically we delineated the exact structure of the mental healthcare system and the existing legal mechanisms of partnerships. As a result, to the best of our knowledge we provide the first description of exact structures and flow of the regional and local mental health care system network in the region of the XIII RHD that may serve as a guide for understanding healthcare system in other parts of Brazil. Moreover, in the process of the system development and implementation, we have received a great deal of support from municipal and state health authorities through regional health committees and advisory groups, which were pivotal to both acquisition of the required technology and the application of the SISAM13 in daily clinical practice [15, 17].

The main difficulties encountered during the implementation and development of the SISAM-13 were: (a) to harmonize both the demands of clinicians involved in direct patient care and the demands of managers of services and service networks; (b) ensure adequate access to the internet in all services; (c) guarantee at least one computer permanently connected to the internet in each service to allow the use of the system; (d) ensure a continuous and active user collaboration to monitor and improve the system through various strategies (monitoring group, survey and periodic training); e) ensure the availability of a team of computer technicians capable of developing the software.

The main current limitation of the SISAM-13 is that it is mostly used only to manage the interface between hospital and community services (i.e. hospitalizations and discharges). Other functions that are available through the system—such as record-keeping of an outpatient clinic appointment—are underused.

The CIIS has received additional funding from the Secretary of Health of Sao Paulo state, for further maintenance, improvement and expansion of the scope of the SISAM13. The system has been presented to managers of regional health departments of Sao Paulo state adjacent to XIII RHD, who have requested support for the system implementation and this request will be presented to the State Health Secretariat, along with detailed request for additional resources needed for expansion. To implement the system in other regions of Brazil the following minimum requirements need to be met: (a) availability of stakeholders willing to participate in meetings aiming for the system adaptation/implementation; (b) provision of the minimum human resources and equipment and (c) provision of adequately trained and funded system maintenance staff. The system will be further evaluated and improved by encouraging more users to complete feedback surveys and by conducting a qualitative research project. To achieve a more complete description of the network operation through health indicators, we developed an adaptation of the mental health matrix model [14, 18], for use as a conceptual framework to guide the development of a basic electronic health record that allows specific adaptations for every type of service [10–12, 17, 19].

## Abbreviations

XIII RHD: The Regional Health Department
CSS: cascading style sheets
ECN: the emergency care network
GHU: The General Hospital of University of Sao Paulo
HTML: hypertext markup language
ICD-10: The International Statistical Classification of Diseases and Related Health Problems
MHSN: the public mental healthcare service network
MHSSN: the mental health specialized services network
PHP: hypertext pre processor
RPMS-USP: The Ribeirao Preto Medical School of the University of Sao Paulo
SD: standard deviation
‘SISAM 13′: Mental Health Information System of the XIII Regional Health Department
